# Does Preputial Reconstruction Increase Complication Rate of Hypospadias Repair? 20-Year Systematic Review and Meta-Analysis

**DOI:** 10.3389/fped.2016.00041

**Published:** 2016-04-28

**Authors:** Marco Castagnetti, Michele Gnech, Lorenzo Angelini, Waifro Rigamonti, Vincenzo Bagnara, Ciro Esposito

**Affiliations:** ^1^Section of Paediatric Urology, Urology Unit, University Hospital of Padova, Padua, Italy; ^2^Pediatric Surgery, Institute for Maternal and Child Health, IRCCS “Burlo Garofolo”, Trieste, Italy; ^3^Department of Paediatric Surgery, Policlinico “G.B. Morgagni”, Catania, Italy; ^4^Department of Paediatrics, Federico II University of Naples, Naples, Italy

**Keywords:** hypospadias, preputial reconstructrion, phimosis, dehiscence, complication

## Abstract

**Introduction:**

We performed a systematic review of the literature on preputial reconstruction (PR) during hypospadias repair to determine the cumulative risk of preputial skin complications and the influence of PR on urethroplasty complications, namely, fistula formation and overall reoperation rate of the repair.

**Materials and Methods:**

A systematic search of the literature published after 06/1995 was performed in 06/2015 using the keyword “hypospadias.” Only studies on the outcome of PR in children, defined as dehiscence of the reconstructed prepuce or secondary phimosis needing circumcision, were selected. A meta-analysis of studies comparing PR vs. circumcision was performed for the outcomes “hypospadias fistula formation” and “reoperation rate.”

**Results:**

Twenty studies were identified. Nineteen reported the outcome of PR in 2115 patients. Overall, 95% (2016/2115) of patients undergoing PR had distal hypospadias. The cumulative rate of PR complications was 7.7% (163/2115 patients), including 5.7% (121/2115 patients) preputial dehiscences and 1.5% (35/2117 reported patients) secondary phimoses needing circumcision. A meta-analysis of seven studies comparing patients undergoing PR vs. circumcision showed no increased risk of urethral fistula formation associated with PR, odds ratio (OR) (Mantel–Haenszel, Fixed effect, 95% CI), 1.25 (0.80–1.97). Likewise, two studies comparing the overall reoperation rate did not show an increased risk of reoperation associated with PR, OR (Mantel–Haenszel, Random effect, 95% CI), 1.27 (0.45–3.58).

**Conclusion:**

PR carries an 8% risk of specific complications (dehiscence of reconstructed prepuce or secondary phimosis needing circumcision), but does not seem to increase the risk of urethroplasty complications, and the overall reoperation rate of hypospadias repair.

## Introduction

Preputial asymmetry is one of the components of hypospadias. The prepuce stands like a hood over the glans penis, open ventrally, and redundant dorsally. Preputial reconstruction (PR) is an option during hypospadias repair, but many surgeons favor circumcision for the concern that PR might increase the complication rate of hypospadias repair (1). PR might do so because it carries specific complications, namely, dehiscence of the reconstructed prepuce and secondary phimosis, but the concern also exists that it might jeopardize the urethroplasty thereby increasing the risk of fistula formation (1). Nevertheless, the range of reported rates of specific preputial complications after PR is wide, with rates being completely negligible in some series (2), and it is unclear whether the arguments concerning the association between PR and urethroplasty complications are based on evidence or merely theoretical.

We performed a systematic review of the literature on PR during hypospadias repair to determine the cumulative risk of specific complications related to the procedure. Moreover, we performed a meta-analysis of studies comparing urethroplasty complications and reoperation rates in patients undergoing hypospadias repair associated with PR vs. circumcision to test the hypothesis that circumcision is associated with a lower rate of complications related to the urethroplasty, namely, fistula rate, and a lower reoperation rate.

## Materials and Methods

A systematic review of the literature published after June 1995 was performed in June 2015 in agreement with the PRISMA statement (3). Three databases, namely, MEDLINE/PubMed, Scopus, and The Cochrane library, were searched for using the free text “hypospadias repair,” in all fields of the records for MEDLINE/PubMed search, and in the Title and Topic fields for the Web of Science and Cochrane library searches. For MEDLINE/PubMed search, “English,” “Humans,” “Males,” and “Publication date from June 1995 to June 2015” were considered, as limits. Subsequently, the queries were pooled without applying any limit.

One-thousand two-hundred thirty records were retrieved by searching MEDLINE/PubMed, 2461 Web of Sciences, and 1 the Cochrane library. Three doctors reviewed separately the abstracts. Papers relevant to the topic of the review were selected by consensus. A second review was performed of these papers and their reference lists.

In the review, we included only studies reporting data on complications of PR (dehiscence of reconstructed prepuce and phimosis), on urethroplasty complications (fistula formation), and on reoperation rates in patients undergoing hypospadias repair with preservation of the prepuce. Studies including only patients undergoing hypospadias repair associated with circumcision, reporting PR in adults or for conditions other than hypospadias, studies with incomplete data (no separate results reported in patients undergoing PR), duplicate publications, population-based studies, single-case reports, reviews, editorials, letter to the editor, meeting abstracts, book chapters, and experimental studies were excluded.

For data extraction, dehiscence was considered irrespective of whether partial or complete. Since many reconstructed prepuces are non-retractile soon after surgery, but retractility can spontaneously improve over time, secondary phimosis was defined as a phimosis needing circumcision during follow-up.

Papers were categorized according to the Oxford Level of Evidence Working Group 2011 levels of evidence (LOEs) for therapy studies including LOE 1, systematic review of randomized trials; LOE 2, randomized trial or observational study with dramatic effect; LOE 3, non-randomized controlled cohort or follow-up study; LOE 4, case series, case–control study, or historically controlled study; or LOE 5, mechanism-based reasoning (4).

Data analysis was subdivided into two parts. First, we performed a cumulative analysis of complications of PR in the reported series. We assessed the overall complication rate and the rate of two specific complications of PR, namely, preputial dehiscence and secondary phimosis. Second, a meta-analysis of case–control and randomized studies comparing PR vs. circumcision was performed. Two different outcomes were used for the meta-analysis, namely, “hypospadias fistula formation” and “overall reoperation rate.” The analysis was conducted using Review Manager v5.2 software designed for composing Cochrane Reviews (Cochrane Collaboration, Oxford, UK). Statistical heterogeneity was tested using the chi-square test. A *p* value <0.10 was used to indicate heterogeneity. If there was a lack of heterogeneity, fixed-effects models were used for the analysis. Random-effects models were used in cases of heterogeneity. Odds Ratios (OR) and 95% confidence intervals (OR 95% CI) were calculated to determine the influence of PR on the selected outcome.

## Results

**Figure 1:**
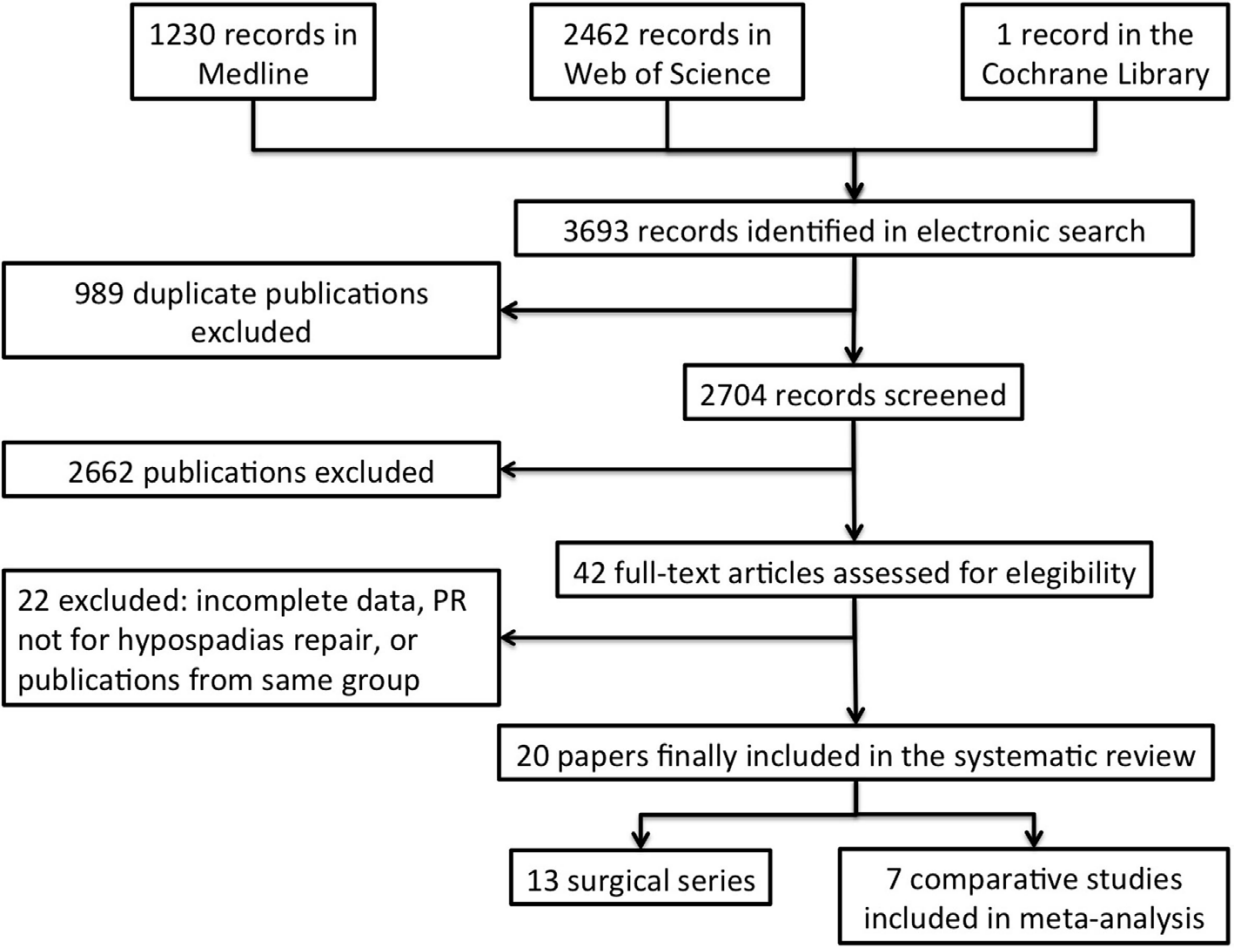
**Flowchart showing the process for selection of studies included in the systematic review**.

Of the original 3692 records, 20 (0.6%) studies that matched the criteria for inclusion in the review were finally selected (Figure 1). Characteristics of included studies are detailed in Table 1. The vast majority (13, 65%) were surgical series (LOE 4), three (15%) were retrospective case–control studies (LOE 4), two (10%) were longitudinal cohort studies (LOE 3), and the remaining two (10%) were RCTs (LOE 2). Also the latter, however, were fraught with significant methodological bias such as lack of power analysis, unclear randomization method, and/or lack of blinding. Studies originated from many different countries both European and non-European (Table 1). The 20 studies included 2215 patients undergoing preputial sparing hypospadias repair. Accurate data about the actual proportion of hypospadias repairs performed at each institution undergoing PR could not be extrapolated, but the rate ranged between 11 and 85%. Only one case series (LOE 4) reported PR in patients with hypospadias associated with ventral curvature (2), whereas 96% (2016/2115) of reported patients undergoing PR had distal hypospadias without associated curvature. PR was generally performed in association with a tubularized incised plate urethroplasty (TIPU), a Mathieu flip-flap urethroplasty, or some kind of glanuloplasty. Two series reported on the use of isolated PR (or in association with a meatotomy) as treatment of hypospadias (5, 6).

**Table 1:** **List of studies (*n* = 20) include in the review**.

Author	Country of origin	Type of study (LOE)	Pts undergoing PR/hypospadias repairs performed (%)	Hypospadias severity (meatal location)	Hypospadias repair
Klijn et al. (7)	The Netherlands	Case series (4)	77/700 (11%)	All distal	Flip-flap urethroplasty or TIPU
Erdenetsetseg et al. (5)	Australia	Case series (4)	51/223 (23%)	All distal	MAGPI 22
					Flip-flap urethroplasty 2
					TIPU 2
					Meatotomy 1
					Nothing 24
Cimador et al. (8)	Italy	Case–control (4)	159/186 (85%)	All distal	MAGPI 22
					Flip-flap urethroplasty 2
Gray et al. (9)	UK	Case–control (4)	205	All distal	All GRAP
Leclair et al. (10)	France	Case series (4)	136/162 (84%)	All distal	All TIPU
Antao et al. (11)	UK	Case series (4)	408	All distal	MAGPI 191
					TPU 171
					Flip-flap urethroplasty 37
Papouis et al. (12)	Greece	Case series (4)	78	All distal	All Flip-flap urethroplasty
Bhatti et al. (13)	UK	Case series (4)	35	All distal	Flip-flap procedure or TIPU
Shimada et al. (14)	Japan	Case series (4)	42/111 (38%)	13 distal	All TIPU
				29 proximal	
Suoub et al. (15)	Canada	Case–control (4)	25/215 (12%)	All distal	All TIPU
Korvald et al. (16)	Norway	Case series (4)	100/122 (82%)	116 distal	All TIPU
				6 proximal	
Hayashi et al. (2)	Japan	Case series (4)	9	All distal	All TIPU
Bhat et al. (17)	India	Case–control (4)	27	All proximal	All TIPU
Fasching et al. (18)	Austria	Case–control (4)	33/64 (51%)	Not specified	All flip-flap procedures
Moslemi et al. (19)	Iran	RCT (2)	43	All distal	All TIPU
ElGanainy et al. (20)	Egypt	RCT (2)	100	All distal	All Flip-flap urethroplasty
Kallampallil et al. (21)	UK	Prospective (3)	218/278 (78%)	170 distal	Anatomical reconstruction
				37 proximal	
Snodgrass et al. (22)	USA	Prospective (3)	85/428 (20%)	All distal	All TIPU
Esposito et al. (23)	Italy	Prospective (3)	354/445 (79%)	All distal	TIPU 233
					MAGPI 121
Zimmermann and Woodward (6)	UK	Case series (4)	30	All distal	Meatotomy 17
					Nothing 13
Total			2215	99 (4.4%) proximal	

*Studies are sorted by year of publication*.

*TIPU, tubularized icised plate urethroplasty; MAGPI, meatal advancement and glanuloplasty; GRAP, glanular reconstruction and preputioplasty*.

Complication rate of PR was detailed in 19 studies (2115 patients), as a RCT focused only on urethroplasty complications and the prepuce was left untouched during hypospadias repair and removed 6 months after the repair in the absence of urethroplasty complications. In the 19 studies (Table 2), the PR complication rate ranged 0 to 30%, but was <10% in 15. The cumulative rate of PR complications was 7.7% (163 of 2115 patients). The most common complication was preputial dehiscence, which cumulative prevalence was 5.7% (121 of the 2115 patients). Secondary phimosis requiring circumcision occurred in 1.7% (35 of 2117) of patients. It is noteworthy, however, that only 4 of the 19 studies had a mean/median follow-up longer than 24 months and no study reported on preputial retractility after puberty.

**Table 2:** **Complications of preputial reconstruction (PR)**.

Author	No. of Pts	PR complication	Preputial dehiscence	Preputial surgery for phimosis	Follow-up months
Klijn et al. (7)	77	23 (30%)	23 (30%) (12 partial, 11 complete)	0	30 (15–108)
Erdenetsetseg et al. (5)	51	3 (6%)	2 (4%) both partial	0	12
Cimador et al. (8)	159	16 (9.9%)	6 (3.7%)	10 (6.2%)	45 (14–76)
Gray et al. (9)	205	4 (2%)	0	4 (2%)	NR
Leclair et al. (10)	136	8 (6%)	6 (4.4%)	2 (1.5%)	12 ± 1
Antao et al. (11)	408	42 (10%)	39 (9.5%)	0	11 (1–100)
			All partial		
Papouis et al. (12)	78	5 (6.3%)	2 (2.5%)	1 (1.2%)	12
Bhatti et al. (13)	35	4 (11.5%)	4 (11.5%)	0	14 (6–18)
Shimada et al. (14)	42	2 (5%)	2 (5%)	0	20 (8–32)
Suoub et al. (15)	25	2 (8%)	1 (4%)	1 (4%)	17.5
Korvald et al. (16)	100	18 (15%)	11 (9%)	7 (6%)	NR
Hayashi et al. (2)	9	0	0	0	13 (1–21)
Bhat et al. (17)	27	1 (4%)	1 (4%)	0	18 (6–24)
Fasching et al. (18)	33	8 (24%)	5 (15%)	3 (9%)	56 (16–99)
Moslemi et al. (19)	43	0	0	0	6
Kallampallil et al. (21)	218	6 (6.3%)	2	4	25 (12–56)
Snodgrass et al. (22)	85	2 (2.3%)	0	1 (1.6%, due to BXO)	8 (4–11)
Esposito et al. (23)	354	17 (4.7%)	16 (4.5%)	1 (0.2%)	12
Zimmermann and Woodward (6)	30	2 (6%)	1 (3%)	1 (3%, due to BXO)	<6
Total	2115	163 (7.7%)	121 (5.7%)	35 (1.5%)	

**Figure 2:**
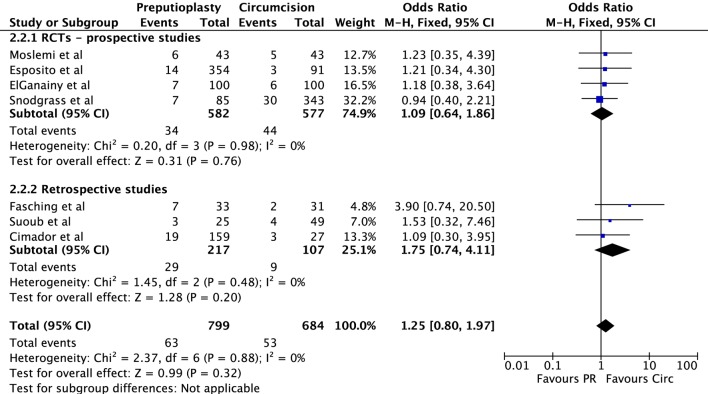
**Forest plot comparing preputioplasty vs. circumcision for the outcome hypospadias fistula formation**.

**Figure 3:**
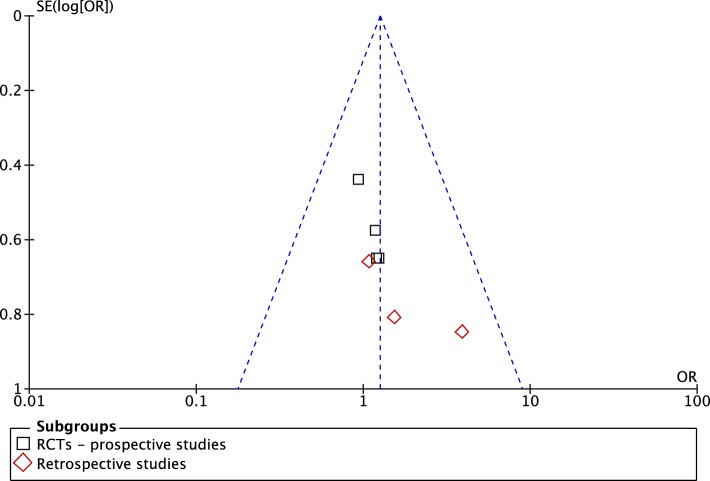
**Funnel plot of comparison: preputioplasty vs. circumcision for the outcome hypospadias fistula formation**.

Seven studies including two RCTs, two prospective longitudinal cohort studies, and three retrospective case–control studies compared the fistula rate in patients undergoing distal hypospadias repair combined with preputial preservation vs. circumcision. A meta-analysis (Figure 2) showed no increased risk of urethral fistula formation in patients where the prepuce was preserved, OR (Mantel–Haenszel, fixed effect, 95% CI), 1.25 (0.80–1.97). This was even more evident after exclusion of retrospective studies, i.e., considering only studies with higher LOE (Figure 2). Funnel Plot did not show evidence of significant bias among studies (Figure 3).

**Figure 4:**
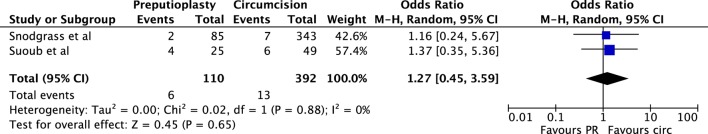
**Forest plot comparing preputioplasty vs. circumcision for the outcome reoperation**.

Only two studies including one prospective longitudinal cohort study and one retrospective case–control study compared overall reoperation rate in patients undergoing distal hypospadias repair associated with PR vs. circumcision. Again, a meta-analysis of these (Figure 4) showed no increased risk of reoperation in patients undergoing PR, OR (Mantel–Haenszel, Random effect, 95% CI), 1.27 (0.45–3.58).

## Discussion

This systematic review shows that according to available evidence PR carries an 8% risk of specific preputial complications, whereas it does not seem to increase the risk of urethroplasty complications and the overall reoperation rate of hypospadias repairs.

Preputial reconstruction is an option during hypospadias repair, and some authors suggest isolated PR to be the procedure of choice in minor variants of hypospadias in order to conceal the malformation while avoiding the risks associated with any urethroplasty (2, 6). The diverse regions of origin of studies included in present review suggests that the procedure is requested all-over the world and Kljin et al. underlined that the request is growing also in centers where circumcision is offered as first option (7). Moreover, studies have shown that preservation of the prepuce is potentially important for the patient and his parents, as the absence of the prepuce is one of the major features that makes patients aware long-term of the surgery they had undergone as children (24–27). Some surgeons, however, are concerned that PR might increase the complication rate of the hypospadias repair because it carries specific skin complications, but also because it might interfere with the healing process of the urethroplasty thereby increasing also the risk of fistula formation (1).

Specific complications of PR include preputial dehiscence and secondary phimosis. The diagnosis of dehiscence is clinical and generally clear-cut. The only problem in the analysis of reported data might concern minor partial dehiscences that could be managed non-operatively. With a prevalence of 6%, dehiscence is indeed the most common complication of PR. It generally occurs soon after surgery. Defining the exact prevalence of secondary phimosis is instead more difficult. The prepuce is commonly non-retractile soon after surgery, but it often becomes retractile during follow-up as the edema subside and the surgical scar mature (1). Additionally, like in children with a physiological phimosis, the prepuce can widen and become fully retractile under the effect of androgens at puberty. Some authors have also recommended the use of steroids to improve preputial retractility, but no control studies are available to determine the role of steroid ointment application compared to spontaneous improvement and simple manipulation. Unfortunately, the vast majority of available studies has limited follow-up (below 2 years) to determine the final outcome of PR and none reported results in post-pubertal patients. Only one study specifically focused on preputial retractility after PR associated with hypospadias repair (21). After a median follow-up of 2.3 years, the prepuce was retractile in 82% (159/194) of patients, 14% (27/194) were under observation for a tight prepuce, and circumcision had been performed for a secondary phimosis in 4% (8/194) of cases. Patients with non-retractile prepuces tended to be younger (median age 5.7 years, range 3–10 years) than those where the prepuce was retractile (median age 7.4 years, range 3–15 years), and the major risk factor for a non-retractile prepuce was the presence of a non-retractile prepuce at the end of surgery (OR 5.97, 95% CI 2.74–13.02). This emphasizes that achievement of a wide and retractile prepuce during surgery is essential and achievement of retractility at the end of surgery should probably be favored over achievement of perfect preputial symmetry (2).

Beside the risk of specific skin complications, a meta-analysis of available data did not show that preserving the prepuce increases urethroplasty complications and particularly fistula formation. This hypothesis was based on the concern that PR impairs use of the prepuce or its pedicle thereof for urethroplasty coverage. Several reasons, however, can otherwise account for the lack of difference in fistula formation between patients undergoing preputial sparing surgery and circumcision. First of all, most of the cases undergoing PR had distal hypospadias, hence the urethral segment reconstructed is relatively short. Second, in such cases, even though a dartos flap is not interposed between the urethroplasty and the skin, the reconstructed urethra is covered by the glans, which is a well-vascularized tissue as well. Third, dartos coverage can anyway be obtained, if desired, just using other kinds of dartos flaps than the dorsal penile tissue (23).

More surprising, the two studies reporting the outcome “reoperation rate” showed no statistical difference also in this outcome between patients undergoing PR and circumcision. This is mainly due to the fact that also circumcision may require revision surgery. This issue is mostly neglected in many hypospadias series and underscores once more, in our opinion, that achievement of adequate skin coverage is sometimes more challenging than that of the urethroplasty itself.

The vast majority of PR was performed in patients with distal hypospadias (96%). It is currently controversial whether PR should be offered only to patients with distal hypospadias and also whether criteria should be defined to select, among distal hypospadias patients, only those with a more favorable preputial anatomy (22). In our opinion, the only absolute contraindication to PR is the presence of ventral curvature due to a ventral skin deficiency (10). Under these circumstances, it is often necessary to transfer the preputial skin ventrally as Byars’ flaps or as an island flap in order to obtain sufficient ventral skin coverage. However, it is true that some cases present with such an asymmetric prepuce that the reconstruction, although feasible, yield extremely poor cosmetic results.

Although this review encompasses the best available evidence, some drawbacks should be considered. Results of systematic reviews are unavoidably dependent on the criteria chosen to select the studies and the quality of the studies included. Regarding the latter, papers included in present review contained only two RCTs and both were fraught with significant methodological limitations. Regarding the criteria for study selection, we included any study about hypospadias repair in its broadest sense, and, as such, also two series of isolated PR. Of course, this procedure does not carry any risk of urethroplasty complications, but might also be associated with a lower rate of specific skin complications because there is less dissection involved in comparison to cases undergoing formal urethroplasty. Nevertheless, we think the concept of isolated PR as treatment of minor hypospadias variants to be important, whereas inclusion of these two series only marginally influenced the cumulative complication rates, every rate increased by only 0.1% after exclusion of these two studies. Moreover, almost all studies have too short follow-up to draw definitive conclusions about the fate of reconstructed prepuces particularly with regards to their retractility after puberty. Still, the review could not account for factors such as the technique used for PR or surgeon experience. Finally, present reviews did not account for the cosmetic outcomes of PR and the importance of PR for the patient and the parents. Unfortunately, data on the latter point are currently scant, and use of validated tools to assess these outcomes is still under scrutiny (23–29); therefore, we considered impossible to analyze these aspects in a structured manner at present.

## Conclusion

Preputial reconstruction is an option particularly in patients with distal hypospadias without associated penile curvature. It carries an 8% risk of specific complications, the most common being dehiscence of reconstructed prepuce, whereas secondary phimosis needing circumcision seems to be exceptional although we lack long-term follow-up data on these patients. Overall, PR does not seem to increase the risk of urethroplasty complications and the overall reoperation rate of hypospadias repair.

## Author Contributions

All authors listed, have made substantial, direct and intellectual contribution to the work, and approved it for publication.

## Conflict of Interest Statement

The authors declare that the research was conducted in the absence of any commercial or financial relationships that could be construed as a potential conflict of interest.
